# Functional implications of the p.Cys680Arg mutation in the MLH1 mismatch repair protein

**DOI:** 10.1002/mgg3.80

**Published:** 2014-05-06

**Authors:** Mev Dominguez-Valentin, Mark Drost, Christina Therkildsen, Eva Rambech, Hans Ehrencrona, Maria Angleys, Thomas Lau Hansen, Niels de Wind, Mef Nilbert, Lene Juel Rasmussen

**Affiliations:** 1 Department of Oncology, Institute for Clinical Sciences, Lund University22185, Lund, Sweden; 2 The Danish HNPCC-register, Clinical Research Centre, Hvidovre University Hospital, Copenhagen UniversityHvidovre, Denmark; 3 Department of Toxicogenetics, Leiden University Medical CenterLeiden, The Netherlands; 4 Department of Clinical Genetics, University and Regional Laboratories, Skåne University Hospital, Lund UniversityLund, Sweden; 5 Center for Healthy Aging, Department of Cellular and Molecular Medicine, University of CopenhagenDK-2200, Copenhagen N, Denmark

**Keywords:** Functional assay, Lynch syndrome, mismatch repair, *MLH1*

## Abstract

In clinical genetic diagnostics, it is difficult to predict whether genetic mutations that do not greatly alter the primary sequence of the encoded protein causing unknown functional effects on cognate proteins lead to development of disease. Here, we report the clinical identification of c.2038 T>C missense mutation in exon 18 of the human *MLH1* gene and biochemically characterization of the p.Cys680Arg mutant MLH1 protein to implicate it in the pathogenicity of the Lynch syndrome (LS). We show that the mutation is deficient in DNA mismatch repair and, therefore, contributing to LS in the carriers.

Lynch syndrome (LS) is a multi-tumor syndrome with particularly high risks of colorectal, endometrial, and ovarian cancers. The syndrome is caused by germline DNA mismatch repair (MMR) gene mutations with major contributions from *MLH1* (MIM#120436) (42%), *MSH2* (MIM#609309) (33%), *MSH6* (MIM#600678) (18%), and *PMS2* (MIM#600259) (8%) (Plazzer et al. 2013). Missense variants are estimated to represent one third of the alterations and frequently pose problems in genetic diagnostics related to their functional consequence and possibilities to apply in predictive diagnostics (Peltomaki and Vasen 2004; Nilbert et al. 2009; Fan et al. 2012). While some of these have been defined as disease predisposing, the pathogenic importance of others remains to be defined (Takahashi et al. 2007; Drost et al. 2010). Such variants of uncertain significance (VUS) represent a challenge for clinicians and genetic counselors because of their undefined consequences (Heinen and Juel Rasmussen 2012; Rasmussen et al. 2012).

We identified a missense mutation *MLH1* (NM_000249.3:c.2038 T>C, dbSNP rs63750809) in genetic diagnostics and present evidence for its causality for LS. The individual tested had developed four LS-associated tumors: an endometrial cancer with an ovarian metastasis at age 50; a right-sided colon cancer, T3N0, at age 51; and two synchronous colorectal cancers, a T3N0 cancer of the transverse colon and a T4N0 rectal cancer, at age 67. The family history was limited to a father with a malignant melanoma at age 88. First-step assessment (Data S1) revealed defective MMR, expressed as microsatellite instability (MSI) for the markers BAT-25, MONO-27, NR-21, and NR-24, immunohistochemical loss of MLH1/PMS2, and wild type for *BRAF* (V600E) mutation in all four tumors (Fig. 1). Mutation analysis of MMR genes in lymphocytes identified a missense mutation in *MLH1*, c.2038 T>C. The variant has been described as pathogenic in silico (Beroud et al. 2000, 2005; Frederic et al. 2009; Ali et al. 2012), and as having unknown effect (Sheng et al. 2008; Thompson et al. 2013). The c.2038 T>C alteration leads to a p.Cys680Arg amino acid substitution that is located in the region of MLH1 that is essential for interaction with PMS2 (Guerrette et al. 1999). In silico analysis was performed using the Polyphen, MAPP-MMR (http://mappmmr.blueankh.com), SIFT (http://sift.jcvi.org), and P-mut methods, which predict the impact of mutations on protein function based on evolutionary conservation using sequence-based information (Ferrer-Costa et al. 2004; Chao et al. 2008; Kumar et al. 2009; Adzhubei et al. 2010; Ali et al. 2012). All of these models predicted that the variant affects protein function (Table 1).

**Table 1:** Summary of biochemical, in silico, and functional analysis of the p.Cys680Arg

Clinical information	Clinical criteria	Amsterdam II
	Age at onset	50
Biochemical analysis	Microsatellite instability	MSI high
	Immunohistochemical staining	MLH1-/PMS2-
In silico analysis	Polyphen	0.970 (probably damaging)
	SIFT	0.02 (not tolerated)
	MAPP-MMR	34.150 (affect protein function)
	P-mut	0.7897 (pathological)
Functional analysis	Cell-free mismatch repair	Pathogenic
	Yeast two-hybrid	No interaction with PMS2

**Figure 1:**
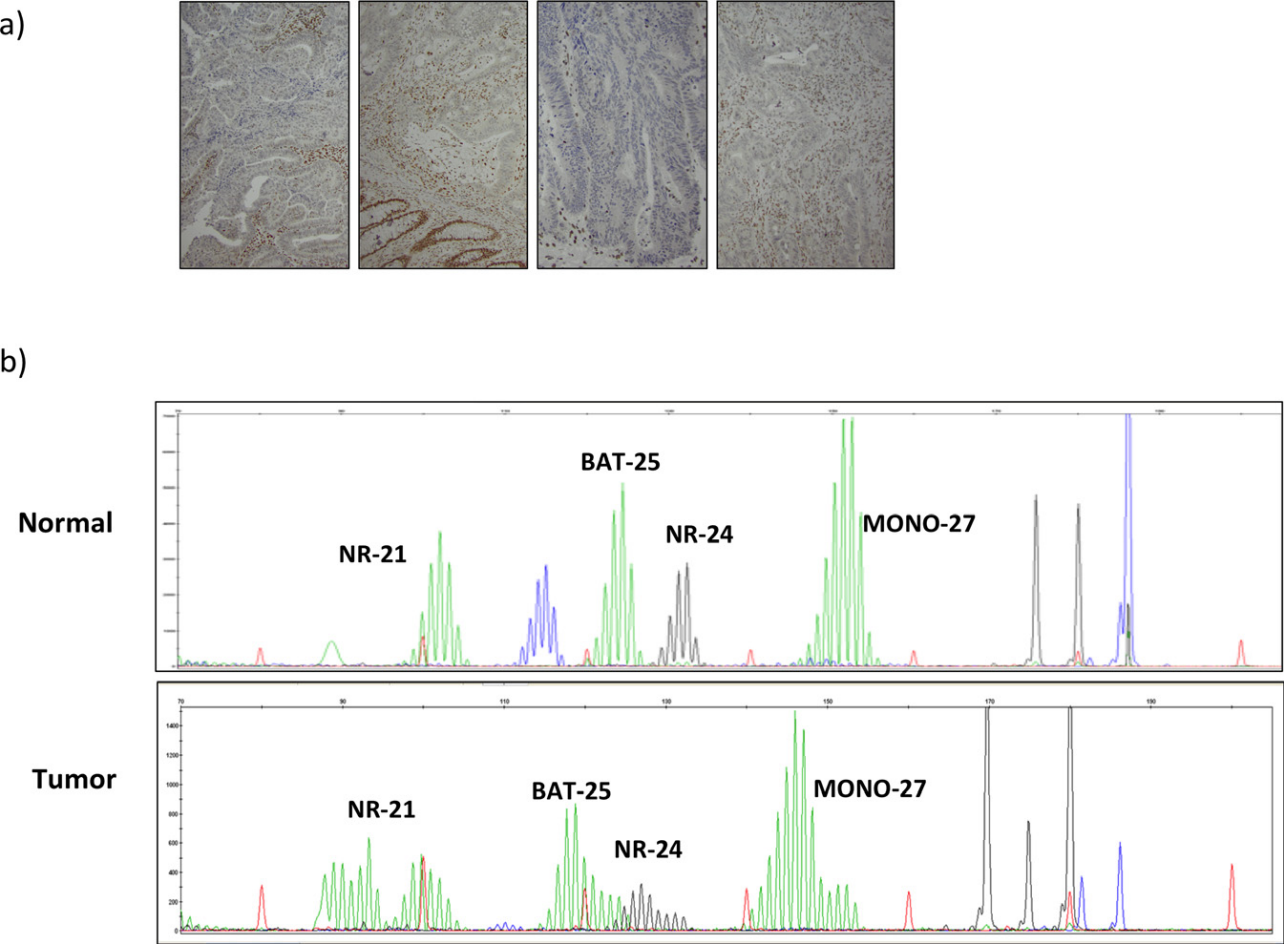
(A) Immunohistochemical staining for MLH1 in the four different tumors (from left to right, an endometrial cancer with an ovarian metastasis and three colorectal cancers), all of which showed loss of MLH1 staining. (B) Analysis of microsatellite instability (MSI) for the markers NR-21, BAT-25, NR-24, and MONO-27 in the colon cancer at age 67; *x*-axis is size in bases, *y*-axis is fluorescence intensity. Red peaks are internal size standards. Green, blue, and black peaks are amplification products from microsatellite loci. The PCR products from the four amplified microsatellite regions in tumor were compared with the reference normal epithelium. The tumor DNA showed alleles that were not present in the corresponding normal DNA and were classified as MSI positive.

The effect of the p.Cys680Arg mutation on MMR function was tested by assessment of its ability to repair a G·T mismatched substrate in a cell-free mismatch repair assay (Drost et al. 2010) (Fig. 2A). Protein variants p.Gly67Arg (c.199G>A) and p.Ile219Val (c.655A>G) were included as controls (Drost et al. 2010). The low repair level observed with the variant p.Gly67Arg reflects its pathogenicity. Variant p.Ile219Val is considered an innocuous polymorphism and showed DNA repair capacity comparable to wild-type MLH1. In this assay, p.Cys680Arg repaired mismatches with an efficiency that was comparable to that of p.Gly67Arg. This strongly supports the pathogenic potential of the p.Cys680Arg variant.

**Figure 2:**
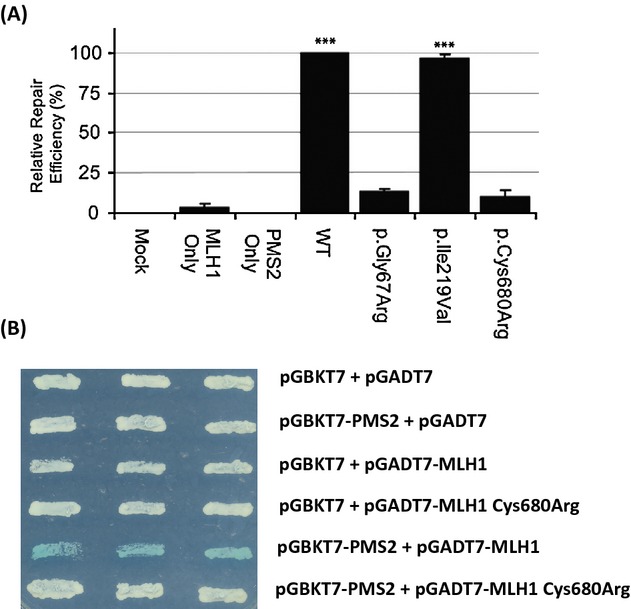
(A) MMR activity of the MLH1 p.Cys680Arg mutant as measured in the in vitro MMR assay. Results are shown as mean ± SEM of four fully independent experiments. Mock, mock expression; Asterisks, significantly higher than established repair-deficient, pathogenic, control p.Gly67Arg (*P* < 0.001, student's one-tailed *t*-test). For “Mock” and “PMS2 only” reactions no repair was detected in all experiments. In vitro MMR assays were performed as described (Drost et al. 2010). In brief, the MLH1 variant alleles were reconstructed by PCR and variant protein was produced in the TnT Quick Coupled Transcription/Translation kit (Promega, Madison, WI), directly from the PCR fragment. Wild-type PMS2 was expressed from a pCITE4a vector (Novagen, Beeston, UK) containing the PMS2 cDNA. Assay reactions were performed in a total volume of 25 *μ*L. Reactions contain 75 *μ*g nuclear extract of the human MLH1 and PMS2 deficient HCT116 colon cancer cell line and 100 ng of substrate (pJHGT3'lnFAM). The cell extract was complemented with 12 *μ*L of in vitro produced MLH1/PMS2 (1:1 v/v). Repair of a T/G mismatch to T/A can be measured after restriction digestion and quantification by fragment analysis. Detected repair efficiencies are normalized to the value for wild-type MLH1. (B) Interaction of MLH1 and MLH1 p.Cys680Arg with PMS2 in the yeast two-hybrid system (Yeast Two-Hybrid Matchmaker Gold, CLONTECH). Strains containing various plasmids were streaked on SD-LEU-TRP + ADE + X-gal plates. The plasmids contained in each strain tested are indicated at the right of each column. The yeast two-hybrid vectors pGBKT7 and pGADT7 (CLONTECH) were used to construct fusion proteins. The yeast two-hybrid assays were performed as accordingly to manufactures guideline (CLONTECH). Briefly, the coding regions of *MLH1* or *MLH1* c.2038 T>C were amplified by PCR using primers recommended by the manufacturer (CLONTECH) and cloned into the *Eco*RI and *Bam*HI sites of pGBKT7 and pGADT7.

The MLH1 protein exists predominately in a complex with PMS2 also known as the MutL*α* heterodimer (Li and Modrich 1995). The formation of a MutL*α* complex is essential for MMR activity (Baker et al. 1995, 1996; Edelmann et al. 1996) and, therefore, the failure of LS-MLH1 proteins to associate with PMS2 could result in inefficient MMR. We show in a yeast two-hybrid assay that the MLH1 p.Cys680Arg variant does not interact with PMS2 suggesting that the MMR-deficiency is caused by failure to form the essential MutL*α* complex (Fig. 2B).

## Summary

The *MLH1* c.2038 T>C mutation, which causes the amino acid substitution p.Cys680Arg, was identified in an individual with four synchronous and metachronous tumors, all of which showed a MSI-high phenotype and loss of MLH1/PMS2 expression. The mutation occurs in a region of the *MLH1* gene that is involved in MLH1-PMS2 interaction. In silico analysis unanimously suggested a deleterious effect of this mutation, which was confirmed by functional assays. We conclude that since the mutation is deficient in MMR it contributes to LS.

## Conflict of Interest

None declared.

## Supporting Information

**Data S1.** Description of methods.
