# Supplementary material for: Functional implications of the p.Cys680Arg mutation in the MLH1 mismatch repair protein
Source: Mol Genet Genomic Med. 2014 May 6;2(4):352–5. doi: 10.1002/mgg3.80 (PMC4113276; doi:10.1002/mgg3.80)
Supplement: Supplementary file 1 — Data S1. Description of methods. [file mgg30002-0352-SD1.docx]

***Immunohistochemistry Analysis***

All tumors were immunostained for the 4 MMR proteins MLH1, PMS2, MSH2 and MSH6. Briefly, 4-µm sections were placed on SuperFrost^®^ Plus microscope slides. Antigen retrieval was performed in a pressure boiler in Target Retrieval Solution, pH 9 (Dako, Glostrup, Denmark) and stained in an automated immunostainer (Autostainer Plus, Dako, Glostrup, Denmark) using Dako EnVision^™^FLEX+ Detection System, Peroxidase/DAB, Rabbit/Mouse (Dako, Glostrup, Denmark), according to the manufacturers' instructions. The antibodies used were MLH1, clone ES05 (Dako, Glostrup, Denmark, dilution 1:100), PMS2, clone A16-4 (BD Pharmingen, San Diego, CA, dilution 1:300), MSH2, clone FE11 (Calbiochem, Merck KgaA, Darmstadt, Germany, dilution 1:100), and MSH6, clone EPR3945 (Epitomics, Burlingame, dilution 1:100). Tumor MMR protein expression was assessed as retained (normal), lost, or weak (i.e. tumor cell staining intensity was reduced compared with that of the normal internal control).

***MSI Analysis***

DNA was extracted from two 10-µm sections using the QIAamp DNA FFPE Tissue Kit (QIAGEN, Valencia, CA). MSI analysis was performed using the MSI Analysis System, Version 1.2 (Promega, Madison, WI) with the products run on a 3130XL Genetic Analyzer (Applied Biosystems, Foster City, CA). The analysis included the 5 mononucleotide markers BAT-25 BAT-26, NR-21, NR-24, and MONO-27 (Promega, MSI Analysis System, Version 1.2, Madison, WI). The results were evaluated using GeneMapper Software Version 4.0 (Applied Biosystems, Foster City, CA) and defined as MSI high when ≥2 markers were unstable, MSI low when 1 marker was unstable and MSS when none of the markers were unstable.

***Mutation analysis***

Genomic DNA was extracted from EDTA blood samples using the QIAamp DNA Mini Kit according to manufacturer´s instructions. Mutation screening was performed for *MLH1* and *PMS2* genes by sequencing of all exons including the intron–exon boundaries using ABI 377 or ABI 3130 DNA sequencers (Applied Biosystems, Foster City, CA) followed by analysis for large intragenic deletions using multiplex ligation-dependent probe amplification (MRC- Holland, Amsterdam, the Netherlands) according to the manufacturers’ instructions.
